# Preliminary results from the LUX‐Dx insertable cardiac monitor remote programming and performance (LUX‐Dx PERFORM) study

**DOI:** 10.1002/clc.23930

**Published:** 2022-10-08

**Authors:** Craig Stolen, Jonathan Rosman, Harish Manyam, Brian Kwan, Jonathan Kelly, David Perschbacher, John Garner, Mark Richards

**Affiliations:** ^1^ Cardiac Rythm Management Boston Scientific St Paul Minnesota USA; ^2^ Cardiac Arrhythmia Service Florida Atlantic University CESCOM Boca Raton Florida USA; ^3^ Department of Cardiology, Erlanger Hospital University of Tennessee Knoxville Tennessee USA; ^4^ Department of Clinical Medicine University of Missouri School of Medicine Columbia Missouri USA; ^5^ Yakima Heart Lung & Vascular Clinic Yakima Valley Memorial Yakima Washington USA

**Keywords:** arrhythmia, cryptogenic stroke, device programming, ICM, remote monitoring

## Abstract

Despite the wide adoption of insertable cardiac monitors (ICMs), high false‐positive rates, suboptimal signal quality, limited ability to detect atrial flutter, and lack of remote programming remain challenging. The LUX‐Dx PERFORM study was designed to evaluate novel technologies engineered to address these issues. Here, we present preliminary results from the trial focusing on the safety of ICM insertion, remote monitoring rates, and the feasibility of remote programming. LUX‐Dx PERFORM is a multicenter, prospective, single‐arm, post‐market, observational study with planned enrollment of up to 827 patients from 35 sites in North America. A preliminary cohort consisting of the first 369 patients who were enrolled between March and October 2021 was selected for analysis. Three hundred sixty‐three (363) patients had ICM insertions across inpatient and outpatient settings. The mean time followed was 103.4 ± 61.8 days per patient. The total infection rate was 0.8% (3/363). Interim results show high levels of remote monitoring with a median 94% of days with data transmission (interquartile range: 82–99). Thirteen (13) in‐clinic and 24 remote programming sessions were reported in 34 subjects. Reprogramming examples are presented to highlight signal quality, the ability to detect atrial flutter, and the positive impact of remote programming on patient management. Interim results from LUX‐Dx PERFORM study demonstrate the safety of insertion, high data transmission rates, the ability to detect atrial flutter, and the feasibility of remote programming to optimize arrhythmia detection and improve clinical workflow. Future results from LUX‐Dx PERFORM will further characterize improvements in signal quality and arrhythmia detection.

## 1 INTRODUCTION

Insertable cardiac monitors (ICMs) have become widely adopted in clinical electrophysiology practice. Their utility in the diagnosis of arrhythmias in clinical contexts such as syncope, cryptogenic stroke, and rhythm management before and after ablation has been well established. They have been shown to determine causation and impact management in significant numbers of patients.^1^, ^2^, ^3^ However, with increasing use, significant issues regarding ICM performance have become evident. Chief among them have been high percentages of false positives, suboptimal signal‐to‐noise ratios, and poor ability to detect arrhythmias with regular *R–R* intervals, particularly if rates are relatively low.^4^, ^5^, ^6^, ^7^ A large volume of alerts, loss of device connectivity, and staffing issues, including burnout, have consequently created challenges for device clinics.^8^

We have recently published the in silico experience with a new ICM, the LUX‐Dx.^7^ Highlights include improved signal quality, marked reductions in false positives using a two‐step detect‐and‐verify algorithm, an AT algorithm that allows for the detection of regular arrhythmias such as atrial flutter, and the ability to reprogram the device remotely. The latter feature minimizes the need for in‐person visits in device clinics and is particularly attractive in the ongoing COVID pandemic environment.^9^

The LUX‐Dx PERFORM trial is the in vivo follow‐up to our previous study and is designed to evaluate the safety of the ICM insertion, the performance of arrhythmia detection algorithms, and the feasibility of remote programming. In this report, we evaluate the safety of ICM insertion, remote monitoring rates, and the feasibility of remote programming in the first cohort of patients enrolled in the LUX‐Dx PERFORM trial.

## 2 METHODS

### 2.1 Study design

The LUX‐Dx PERFORM Study is multicenter, prospective, single‐arm, post‐market, observational study (ClinicalTrials. gov Identifier: NCT04732728). The trial is being conducted at up to 35 sites in the US and will enroll approximately 600 to 827 subjects. A cohort consisting of the first 369 enrolled patients was selected for interim reporting. The investigation conforms to the principles outlined in the Declaration of Helsinki, Institutional Review Board approval has been obtained at each center, and all patients provided written informed consent.

### 2.2 Study population and eligibility

Subjects are selected from the investigators' patient population per labeled indication for LUX‐Dx ICM insertion. The LUX‐Dx ICM System is indicated to monitor and record Subcutaneous ECGs for the clinical evaluation and diagnosis of cardiac arrhythmias in patients who have: (1) a known heart condition, (2) are at risk of developing an abnormal heart rhythm, or (3) have symptoms that may suggest a cardiac arrhythmia (e.g. dizziness, palpitations, syncope, chest pain, and/or shortness of breath). All patients who meet all the inclusion criteria and none of the exclusion criteria may be given consideration for participation (Supporting Information: Table 1).

### 2.3 Study system

The LUX‐Dx ICM (Boston Scientific Corporation) uses a data management system, LATITUDE Clarity™, which allows clinicians to remotely monitor and program the ICM. To transfer the ICM device data to LATITUDE Clarity, the system utilizes the myLUX™ Patient mobile app and the LUX‐Dx Clinic Assistant mobile app on dedicated smartphones. This allows clinicians to access the device data for analysis to support their evaluation of the clinical status and to adjust the diagnostic programmable features as needed (Supporting Information: Figure 1).

### 2.4 Study visits

The ICM insertion procedure must be performed within 30 days following informed consent. Relevant study data including insertion procedure‐related data, reportable adverse events, and ICM device data from the LATITUDE Clarity, is being collected during the subject's study participation period. Specifically, participating sites review each enrolled subject's electronic medical record (EMR) and communicate with the subject via telephone calls/virtual health visit or in‐office visit at two primary time points (6 and 12 months after insertion) for documentation of relevant adverse events. For this study, these follow‐up visits do not require subjects' physical presence at the study site. A cohort of approximately 600 subjects will wear an extended Holter monitor (HM) for approximately 14 days. The HM visit will be completed 1–3 months postinsertion. This, combined with formal adjudication of these rhythm strips, will allow for calculation of ICM diagnostic performance.

### 2.5 Primary objectives

The primary objective of this interim analysis is to characterize the ICM remote monitoring rates and the utilization of the remote programming feature. Future results from LUX‐Dx PERFORM will characterize the performance of the arrhythmia detection algorithms in the LUX‐DX ICM, as well as report the ICM system‐related complication‐free rates, for the full study population, at 30 days 12 months postinsertion.

### 2.6 Statistical methods and sample size calculations

The interim analysis objectives do not have formal statistical hypotheses. Performance of the arrhythmia detection algorithm will be evaluated at the end of the study by calculating the positive predictive value (PPV) of arrhythmias captured by the ICM, using adjudicated HM tracings as the reference standard. ICM system‐related complication‐free rates will be assessed using Kaplan‐Meier methodology comparing the rate and one‐side pointwise log‐log confidence interval with a performance criterion of 94% at 30 days and 93% at 12 months.

## 3 PRELIMINARY RESULTS

### 3.1 Study population

First enrollment was on March 5, 2021 and as of October 14, 2021, 369 patients were enrolled in the LUX‐Dx PERFORM study and 363 patients had device insertions at 18 centers in the United States (Supporting Information: Figure 2). The mean time followed in the study was 103.4 ± 61.8 days (range 1–223 days) per patient. The primary indications for insertion were cryptogenic stroke 91 (24.7%), syncope 112 (30.4%), and the remainder Rhythm Management (44.9%), The mean age was 65.8 ± 14.6 years and 50% were male (Table 1). Patients had a history of ventricular arrhythmias 71(19.2%), atrial arrhythmias 197 (53.4%), and brady arrhythmias 75 (20.3%) at the time of enrollment (Supporting Information: Table 2). The ICM insertion procedure was performed in‐hospital 77% of the time (*N* = 279) and in‐clinic 23% of the time (*N* = 83).

**Table 1:** Baseline characteristics

Characteristic	Measure	All patients (*N* = 369)^a^
Age (years)	*N*	369
	Mean ± SD (Med)	65.8 ± 14.6
	Range	(19.0–94.0)
Sex	Male	185 (50.1%)
Race^b^	White	334 (92.8%)
	Black or African American	24 (6.7%)
	Asian	2 (0.6%)
	Other	1 (0.3%)
Height (cm)	*N*	368
	Mean ± SD (Med)	170.9 ± 10.5
	Range	(132.1–198.1)
Weight (kg)	*N*	368
	Mean ± SD (Med)	88.7 ± 23.8
	Range	(44.4–208.7)
Comorbidities	COPD	26 (7.1%)
	Syncope	137 (37.2%)
	Diabetes	86 (23.4%)
	Cerebrovascular disease	75 (20.4%)
	Cryptogenic stroke	48 (64.0%)
	Peripheral vascular disease	21 (5.7%)
	Renal dysfunction	37 (10.1%)
	Hypertension	263 (71.5%)
	Hyperlipidemia	206 (56.0%)
	Anemia	24 (6.5%)
	Sleep apnea	77 (20.9%)
History of disease/risk factors^b^	Heart failure	42 (11.4%)
	Ischemic heart disease	39 (10.6%)
	Valvular disease	45 (12.2%)
	COVID‐19	13 (7.4%)

^a^ Percentages calculated out of total patients with non‐missing values.

^b^ Patients may contribute to more than one measure.

Seven adverse device events were reported. One patient (1; 0.3%) had placed a Tegaderm over the incision site when taking a shower and the skin glue came off when the Tegaderm was removed. The incision was reglued and there were no signs of infection. One patient (1; 0.3%) returned the day of insertion with incision site bleeding, which resolved with pressure, epi/lidocaine injection, nonabsorbable sutures, and redressing. Three patients had infections (3; 0.8%); two were treated with oral antibiotics for infections that occurred ≤10 days post in‐hospital insertion and resolved without further mitigation. The third patient had an infection 50 days post in‐hospital insertion that required treatment with oral antibiotics and ICM explant. There was also one report of ICM erosion and removal (1; 0.3%), the site was cleaned, and oral antibiotics were ordered for erythema. One patient (1; 0.3%) developed a rash from a Holter patch that resolved with steroid cream.

### 3.2 Connectivity

**Figure 1:**
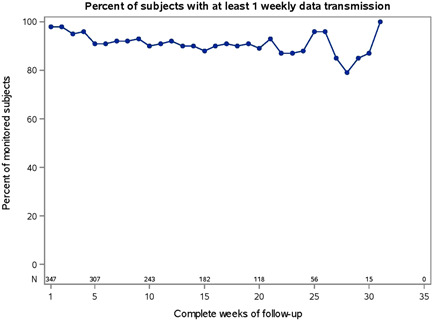
Remote monitoring rates. Percentage of patients with at least 1 weekly Lux‐Dx data transmission.

One concern with using Bluetooth communication as the means of data transfer is that failure to maintain connectivity may occur when mobile devices are out of battery power, or when patients go for extended periods without their mobile device on their person. In this cohort, successful daily data transmission from the ICM to LATITUDE Clarity occurred 86% of the time (pooled for all patients from the day of first transmission to the day of last transmission) and the median percentage of days with a data transmission for individual patients was 94% (interquartile range: 82–99). Failure to maintain connectivity was uncommon and the percentage of patients with at least 1 weekly data transmission remained high over the course of the monitoring period (Figure 1). The monitoring period started at insertion, was censored at death, withdrawal, or date of data pull (October 14, 2021), and represented a total of 103.2 patient‐years.

### 3.3 Reprogramming

During the evaluation period, multiple ICMs were reprogrammed to optimize arrhythmia detection and diagnosis and to improve clinical workflow. There were 43 reprogramming sessions in 34 subjects (9.4% of the total cohort). Details of 13 in‐clinic and 24 remote sessions were reported by the sites (Table 2). Additional reprogramming sessions were identified through linkage with LATITUDE Clarity Data management system. No untoward consequences from the process of remote ICM reprogramming have been reported to date.

**Table 2:** Programming adjustments and method programming

Parameter name	Values	Parameter adjustments*		Method	
		Increase, *N*	Decrease, *N*	Remote	In‐clinic
Blank after sense	130–140 ms @ 10 ms increments	1		1	
AF response setting	Least, Less, Balanced, More, Most	2	6	7	1
AF duration	2, 4, 6, 10, 20, 30, 60 (min)	1	3	2	2
AT duration	2, 6, 10, 20, 30, 60 (min) and 2, 3, 4, 6, 8, 10, 12, 16, 24 (h)		5	1	4
AT rate	70, 80, 90, 100, 110, 120, 140, 160, 180 (bpm)	1	1	2	
Brady duration	1, 2, 3, 5, 7, 10, 15, 20, 30 (s)	4		4	
Brady rate	30, 40, 50, 60 (bpm)	4	4	4	4
Sensitivity	0.025, 0.037, 0.05, 0.075, 0.1, 0.15, 0.2 mV	2		1	1
Tachy duration	0, 1, 2, 3, 4, 5, 10, 15, 20, 25, 30, 40, 50, 60 (s)	3	1	3	1
Tachy rate	115–120 in steps of 5 bpm	2	8	4	6

*Patients may contribute to more than one category.

The LUX‐Dx *Clinic Assistant app* is intended for use during *in‐clinic follow‐up* and allows for on‐demand connection to any LUX‐Dx ICM, within a range of 2 m. The LUX‐Dx Clinic Assistant app was used five^5^ times to program an ICM by transferring device programming changes from the server to the ICM. The median time from programming adjustments on the LATITUDE Clarity web page to confirmation of receipt by the ICMs was 4.8 min (range 1–31 min), and is inclusive of the time delays between web entry and connection to the patient ICM with the app.

The *myLUX Patient app* is intended primarily for *patient use* and can be used to remotely apply programming changes via scheduled communication or patient‐initiated interrogation. Thirty‐six reprogramming sessions were completed in 24 patients using the myLUX Patient app with an application of changes in a median time of 15 h and 55 min (range 45 min to 5 d 12 h 43 min); 92% (33/36) of the changes were applied within 48 h. Two reprogramming sessions using the myLUX Patient app were in progress at the time of data lock and were not included in the analysis.

#### 3.3.1 Remote programming examples

Two representative examples are presented to illustrate the utility of remote ICM programming.

**Figure 2:**
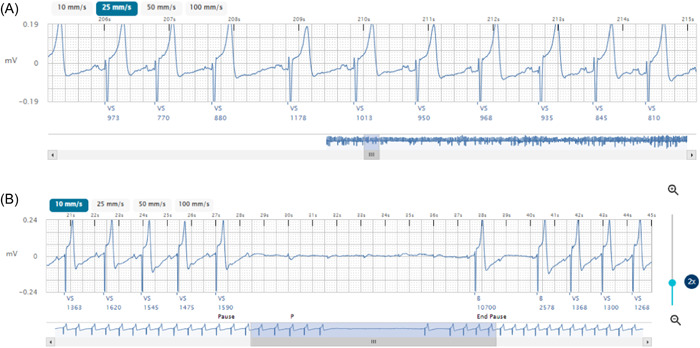
Reprogramming Example 1. (A) False positive episode before remote reprogramming. (B) Ten seconds of complete heart block recognized after reprogramming.

With the first example, an 84‐year‐old gentleman with a prior history of catheter ablation for asymptomatic atrial flutter was referred for an ICM. The patient had been having possible AF episodes according to a commercially available home monitor, but then showed no arrhythmias after a week of monitoring with a wearable cardiac monitor. Post insertion of a LUX‐Dx, a 4‐min‐long “AF” episode was detected with an AF Response setting of “More” and an AF Duration setting of 4 min (nominal settings for Suspected AF). Multiple such episodes were detected. These episodes were reviewed by the electrophysiologist and determined to be sinus rhythm with atrial premature beats and sinus arrhythmia (Figure 2A). The physician remotely reprogrammed the AF Response factor from “More” to “Balanced” to allow the ICM to properly screen out these episodes. This resulted in a marked reduction in false positive episodes: from one every few days to one per month. The ability to remotely reprogram the LUX‐Dx allowed for immediate reprogramming without requiring an office visit. The patient was subsequently found to have 10 s of complete heart block (Figure 2B), for which he underwent pacemaker placement and LUX‐Dx removal.

**Figure 3:**
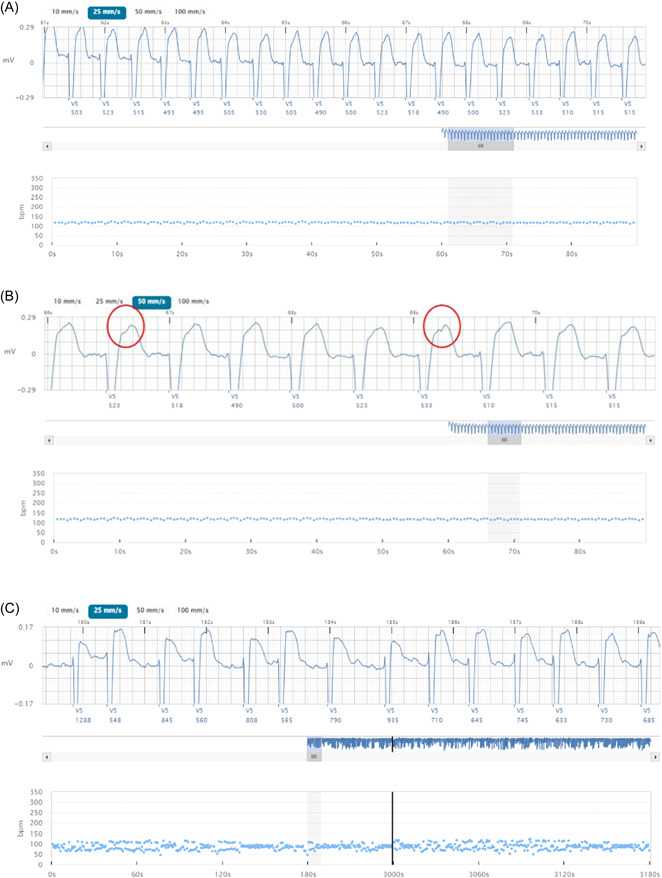
Reprogramming Example 2. (A) Five‐hour episode of regular tachycardia detected by the AT algorithm. (B) Magnification of the ECG illustrating possible atrial flutter. Red circles highlight possible flutter waves. (C) Confirmation of persistent atypical atrial flutter.

In the second example, an 84‐year‐old gentleman with hypertension and diabetes underwent LUX‐Dx ICM insertion for the management of asymptomatic paroxysmal AF. The patient was found to have a 5‐h episode (detected by the AT algorithm; initial settings 110 bpm, 4 h) of a regular tachycardia at 120 bpm, suspicious for atrial flutter (Figure 3A,B). The patient was not symptomatic, and his overall burden of arrhythmia was not significant. However, there was concern that the patient was having sustained atrial flutter below the AT detection rate; therefore, the device was remotely reprogrammed to a lower detection rate (70 bpm). Subsequent AF and AT episodes, after device reprogramming, revealed the patient to be in persistent atypical atrial flutter with a ventricular response of 100–110 bpm and the patient was scheduled for catheter ablation (Figure 3C). Remote device reprogramming and the novel AT algorithm enabled rapid diagnosis and treatment for this patient.

## 4 DISCUSSION

The utility of ICM placement in the diagnosis and management of arrhythmias has now been verified in a variety of clinical contexts.^1^, ^2^, ^3^ Consequently, it is estimated that a quarter million of these devices will be placed in the United States alone in 2022.^10^ However, this huge increase in patient volume has made the limitations of many existing ICMs clearer. The incidence of false positive diagnosis has been documented to be as high as 86%.^4^, ^5^ This is often attributed to poor signal‐to‐noise ratios and low P‐wave voltage. Such false positives increase workload in device clinics significantly. Furthermore, prior algorithms based solely on Lorenz plots and irregularity in the *R–R* interval were ineffective at detecting atrial flutter and were typically programmed off. Finally, the need for reprogramming to attempt to address these performance issues required an on‐site visit absent the ability to reprogram legacy devices remotely.

The LUX‐Dx ICM was designed to address many of these important issues.^7^ Improved signal quality may yield improved algorithm performance and reduced false positives, as well as enhancing the ability of implanting electrophysiologists to interpret complex rhythm strips. Formal analysis in this regard requires rhythm strip adjudication and will be performed at the end of the LUX‐Dx trial. Likewise, the AT algorithm allows for the successful detection of arrhythmias with regular *R–R* intervals. Future results from the LUX‐Dx PERFORM study will evaluate the clinical implementation of these technology advances and will characterize quantitative changes in signal quality and arrhythmia detection.

ICM system‐related complication‐free rates at 30 days and 12 months postinsertion will also be used to demonstrate acute and chronic safety. The interim results of the LUX‐Dx PERFORM trial presented here suggest a favorable safety profile for the LUX‐Dx ICM. As of this analysis, 363 patients had the LUX‐Dx inserted with few adverse device effects. No infections were reported after in‐office procedures and three patients experienced an infection after in‐hospital insertion procedures for a total infection rate of 0.8% (3/363). Two infections resolved with oral antibiotic treatment, and one resulted in explant of the ICM. This low rate of infection is consistent with other reports of low infection rates (0%–1.6%) ^11^, ^12^, ^13^, ^14^, ^15^

The device performance measures reported here affirm in vivo prior findings in an in silico environment. Likewise, transmission rates between the ICM and data management systems have been an issue in the past,^16^ with reported transmission success <80%.^17^ These delays have the potential to impact patient care as only 11.7% of identified life‐threatening events were transmitted from the Medtronic Reveal LINQ™ cardiac monitor within 24 h and 35.8% took longer than 10 days to be transmitted in a 2019 study.^16^ Here, the Bluetooth interface between the LUX‐Dx and the LATITUDE Clarity system, via dedicated smartphones, has been excellent with daily transmissions occurring 86% of the time, and comparable to those achieved with handheld wands and bedside transmitters that report success rates, ranging from 79.5% for the Medtronic Reveal LINQ™^17^ and 92.2%,^18^ 93.8%,^19^ and 94.9%^20^ for the Biotronik BioMonitor 2.

The ability to reprogram these devices remotely has the potential to improve workflow, increase throughput, and reduce the need for clinic visits. The interim results presented here already demonstrate that remote ICM reprogramming is feasible and can positively impact arrhythmia detection and diagnosis.

The two ICM reprogramming examples demonstrate the importance of signal quality and reprogramming ability. In the first example, the ICM was placed for nonspecific complaints of dizziness, palpitations, and possible AF. After remote reprogramming eliminated most false positives, it became clear that intermittent complete heart block was likely responsible for the patient's symptoms. Rather than employing a rhythm control approach which might have worsened his heart block, proper rhythm information allowed identification and treatment of the actual problem. In the second example, the ability of the AT algorithm to accurately detect atrial flutter in vivo was verified. The ability to remotely program in this setting to improve detection was impactful, unmasking the true burden of atrial flutter and abating tachycardia cardiomyopathy risk.

## 5 LIMITATIONS

The presented time periods for reprogramming are real‐world representations of the time from committing programming on the LATITUDE Clarity server to full acceptance and acknowledgment by the device and include circumstantial delays. Examples of such delays include the patient's phone being powered off or with battery depleted, the implanted device being out of range, patients on vacation without phone or service, and so forth.

## 6 CONCLUSIONS

Interim results from the LUX‐Dx PERFORM study demonstrate the use of remote ICM reprogramming to optimize arrhythmia detection and diagnosis, and to improve clinical workflow. Future results from the LUX‐Dx PERFORM study will characterize improvements in signal quality and arrhythmia detection.

## CONFLICTS OF INTEREST

Craig Stolen, Brian Kwan, Jonathan Kelly, and David Perschbacher are Employees of Boston Scientific; Jonathan Rosman and John Garner have no conflicts of interest; Harish Manyam is a consultant for Abbott, Boston Scientific, and Jannssen; Mark Richards is the Principal Investigator of the LUX‐Dx PERFORM study and a consultant for Boston Scientific.

## Supporting information

Supporting information.

## Data Availability

The data from this clinical trial may be made available to other researchers in accordance with Boston Scientific's Data Sharing Policy (https://www.bostonscientific.com/en-US/data-sharing-requests.html).
