# Supplementary material for: Preliminary results from the LUX‐Dx insertable cardiac monitor remote programming and performance (LUX‐Dx PERFORM) study
Source: Clin Cardiol. 2022 Oct 8;46(1):100–7. doi: 10.1002/clc.23930 (PMC9849434; doi:10.1002/clc.23930)
Supplement: Supplementary file 1 — Supporting information. [file CLC-46-100-s001.docx]

**Supplementary Table 1. Inclusion and Exclusion Criteria.**

| **Inclusion Criteria** | • Patient is indicated to be implanted with the LUX-Dx ICM for one of the following reasons (grouped in three “Reason for Monitoring” subgroups):  o Cryptogenic stroke  o Syncope  o AF management, Post-AF ablation, or Suspected AF  • Patient is willing to enroll and be monitored in LATITUDE Clarity.  • Patient is willing and able to be followed remotely via the ICM patient mobile app.  • Patient is willing and capable of providing informed consent (which is not to include the use of a legally authorized representative (LAR) for documentation of informed consent) and agrees to participate in all protocol required activities.  • Patient is age 18 years or above, or of legal age to give informed consent specific to state and national law.  The following inclusion criterion is applicable for patients participating in the Holter study:  • Patient can tolerate the adhesive used in the Holter monitoring for an extended period of time. |
| --- | --- |
| **Exclusion Criteria** | • Patient is indicated for implantation of, or is currently implanted with an active implantable cardiac device (e.g., LVAD, ICD, CRT-D, PPM).  • Patient cannot tolerate a subcutaneous, chronically-inserted device due to medical condition.  • Patient has a documented life expectancy of less than 12 months (per investigator’s discretion).  • Patient is known to be pregnant at the time of study enrollment (method of assessment upon investigator’s discretion).  • Patient is currently enrolled in another clinical study including observational studies/registries, unless prior written approval from BSC is obtained. Mandatory governmental registries are accepted for co-enrollment without approval by BSC.  *The following exclusion criteria are applicable for patients participating in the Holter study:*  • Patient has known allergies to the adhesive materials or hydrogel used in the extended Holter monitoring.  • Patient has broken, damaged, or irritated skin over the chest area where the extended Holter monitor will be attached. |

BSC: Boston Scientific; CRT-D: Cardiac Resynchronization Therapy-Defibrillator; ICD: Implantable Cardioverter Defibrillator; ICM: Insertable Cardiac Monitor; LVAD: Left Ventricular Assist Device; PPM: Permanent Pacemaker

**Supplementary Table 2. Arrhythmia History**

| **Characteristic** | **Measure** | **All Patients (N=369)**** |
| --- | --- | --- |
| History of Ventricular Arrhythmia* | Ventricular Fibrillation (VF) | 0 (0.0%) |
|  | Ventricular Flutter | 0 (0.0%) |
|  | Monomorphic VT (MVT) | 1 (0.3%) |
|  | Polymorphic VT (PVT) | 0 (0.0%) |
|  | Nonsustained VT (NSVT) | 40 (10.9%) |
|  | Torsade de Pointes (TdP) | 0 (0.0%) |
|  | Other Ventricular Arrhythmia | 34 (9.2%) |
| History of Atrial Arrhythmia* | Atrial Fibrillation | 153 (41.6%) |
|  | Atrial Flutter | 58 (15.8%) |
|  | Atrial Tachycardia | 52 (14.1%) |
| Atrial Fibrillation Type | Paroxysmal | 134 (87.6%) |
|  | Permanent | 3 (2.0%) |
|  | Persistent | 16 (10.5%) |
| Atrial Fibrillation Treated with Ablation | Yes | 54 (35.3%) |
| History of Bradyarrhythmia* | Sinus Bradycardia | 52 (14.1%) |
|  | Chronotropic Incompetence | 1 (0.3%) |
|  | AV Block 1 | 10 (2.7%) |
|  | AV Block 2 | 5 (1.4%) |
|  | AV Block 3 | 2 (0.5%) |
|  | Other Bradyarrhythmia | 14 (3.8%) |
| * Patients may contribute to more than one category ** Percentages calculated out of total patients with non-missing values | | |


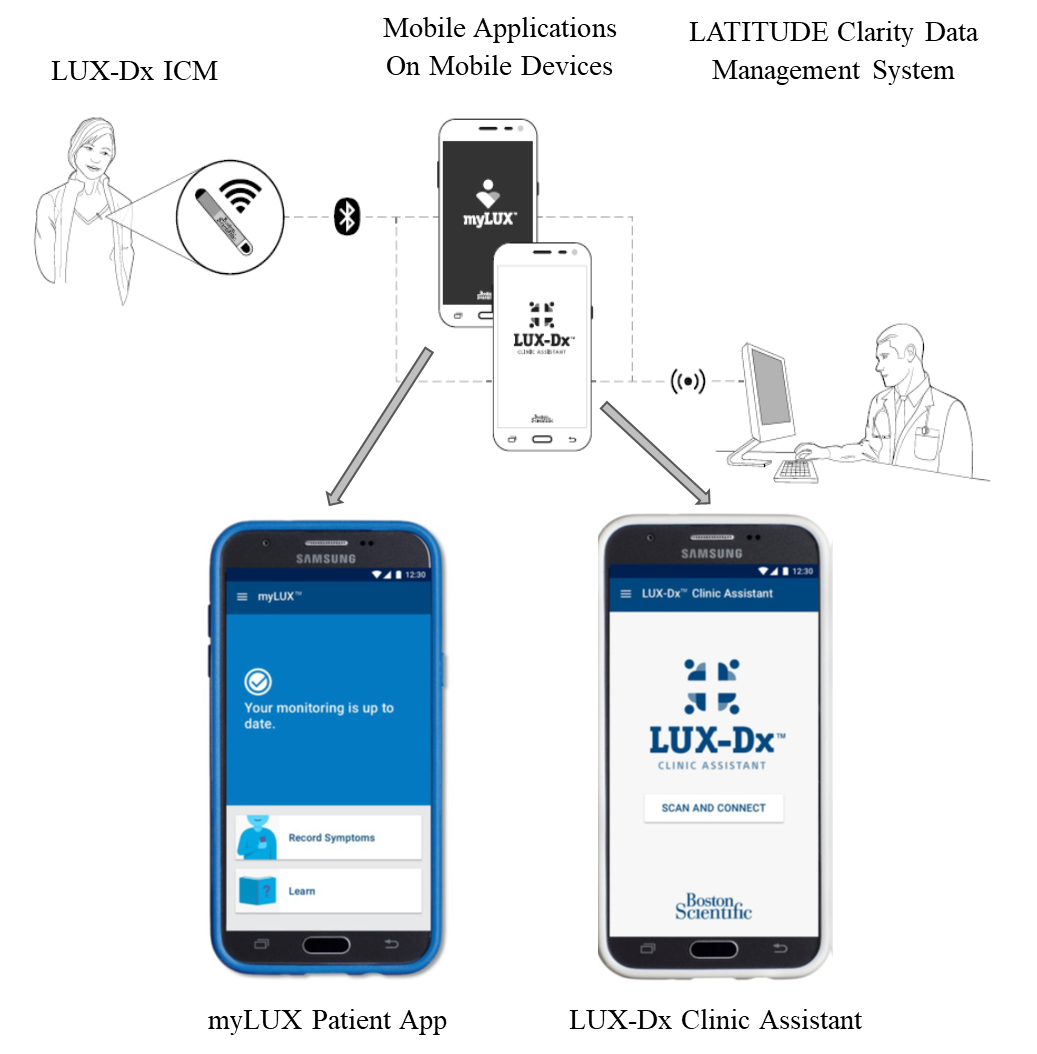
 **Supplementary Figure 1. LUX-Dx ICM System Diagram.**  System components are the LUX-Dx Insertable Cardiac Monitor (ICM), mobile device applications including the myLUX software (patient app) and LUX-Dx Clinic Assistant software (clinic app), and the LATITUDE Clarity Data Server and Website.

*
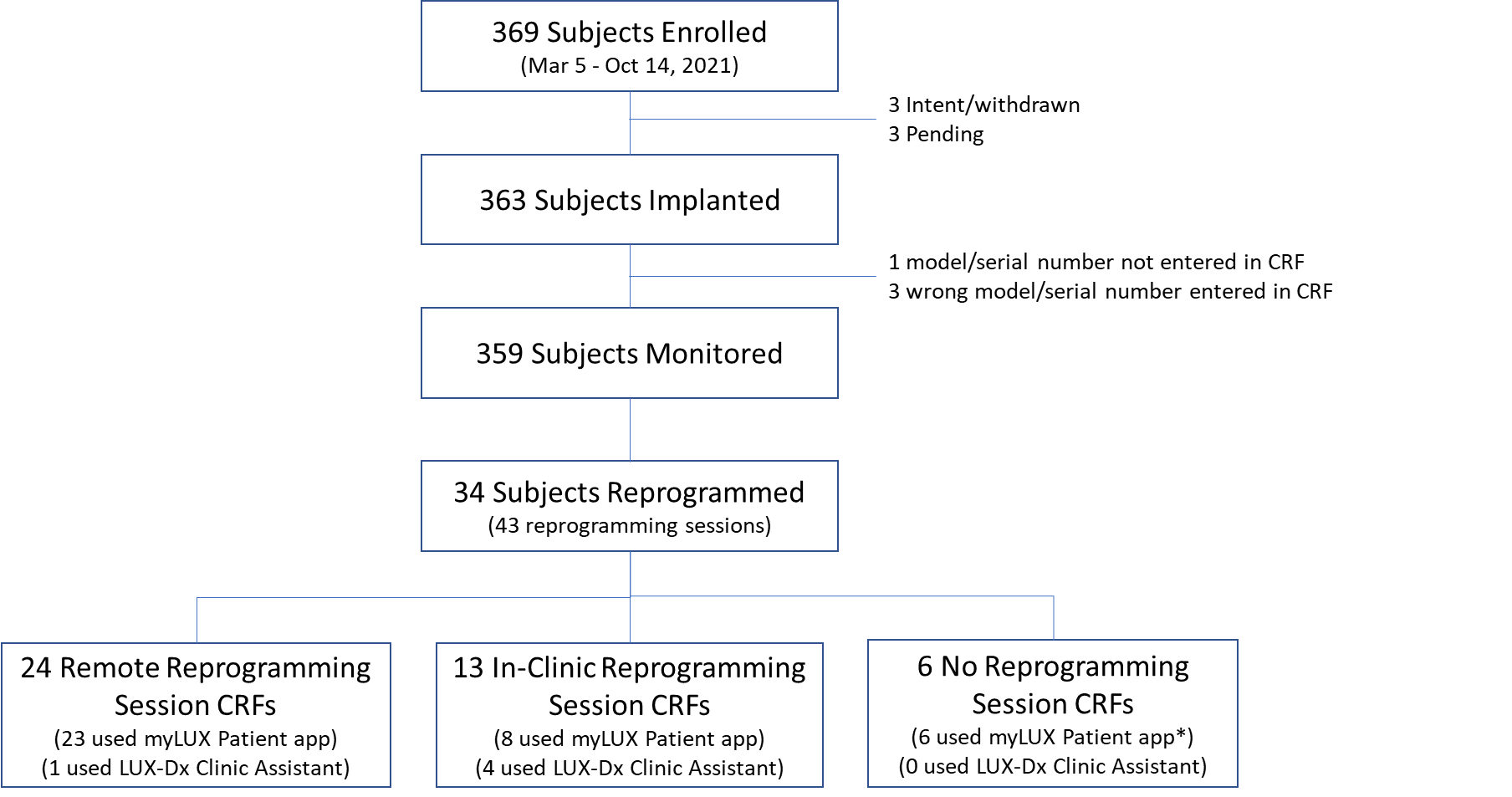
*

**Supplementary Figure 2. LUX-Dx PERFORM Subject Status.** Patient status and reprogramming events as of Oct 14, 2021. * Two reprogramming transmissions were in progress at the time of data lock.
